# ENGAGING OLDER ADULTS TO GUIDE LOW-COST NONWEARABLE SENSOR TECHNOLOGY DEVELOPMENT TO AGE IN PLACE: SURVEY FINDINGS

**DOI:** 10.1093/geroni/igad104.2247

**Published:** 2023-12-21

**Authors:** Elinor Schoenfeld, Tracy Trimboli, Kaylyn Finnerty, Givenchy Ayisi-Boahene, Patricia Bruckenthal, Erez Zadok, Shelley Horwitz, Fan Ye

**Affiliations:** Stony Brook School of Medicine, Stony Brook, New York, United States; Stony Brook University, Stony Brook, New York, United States; Mather Hospital Northwell Health, Port Jefferson, New York, United States; Stony Brook University, Stony Brook, New York, United States; Stony Brook University, Stony Brook, New York, United States; Stony Brook University, Stony Brook, New York, United States; Stony Brook University, Stony Brook, New York, United States; Stony Brook University, Stony Brook, New York, United States

## Abstract

As Americans live longer, a dynamic opportunity has arisen to provide enhanced resources to sustain their well-being. Cost-conscious, convenient in-home sensing will assist with chronic disease management, and become part of a long-term plan to support our aging population and shrinking healthcare workforce. The purpose of this study was to obtain input from older adults about (i) their comfort level and willingness to adopt different sensor technologies, and (ii) opinions on data sharing, security, and privacy to guide our sensor development. Over 4 different survey timeframes (2018-2022), adults aged 60 and older (N=112) completed our survey either in-person (n=77) or via a REDCap online survey (n=35) (53% female; 30% age >80; 78% college graduates; 19% living alone). Though there were significant differences (p< 0.05) in demographics based upon recruitment source, no differences in attitudes towards sensor use were found by age, gender, education, or marital status. Opinions and preferences for sensor type/number/install location, and data sharing preferences significantly differed (p< 0.05) by home living arrangements (independent, 55+ or continuous care communities). Similar to national surveys, changes in technology use were observed pre- versus post COVID. Respondents living in 55+ and continuous-care housing were more comfortable with having sensors installed in their homes than those in community dwelling independent housing. This study highlights the need to include end users throughout the lifecycle of product development and provides insights into preferences by older adults for sensor use and data sharing.

